# 50. Impact of Antibiotic Stewardship Interventions on Colistin Use and Acinetobacter Resistance

**DOI:** 10.1093/ofid/ofab466.252

**Published:** 2021-12-04

**Authors:** Khalid Eljaaly

**Affiliations:** King Abdulaziz University, Jeddah, Makkah, Saudi Arabia

## Abstract

**Background:**

Our hospital had a widespread use of colistin and tigecycline, and very high resistance of Acinetobacter Spp. to colistin. The hospital did not have any infectious disease (ID) pharmacist and had only one ID consultant physician. The objective of this study was to evaluate the impact of our intervention on the utilization of colistin and tigecycline and resistance of Acinetobacter Spp.

**Methods:**

This was a before an observational before-and-after study at a tertiary medical center. An ID pharmacist trained in antibiotic stewardship program (ASP) was invited by a tertiary hospital to help create an ASP. The hospital also hired four ID assistant consultants to help the primary ID consultant and pharmacists. The ASP started by restriction of colistin and tigecycline. The study outcomes were antibiotic consumption and resistance of *Acinetobacter* spp.

**Results:**

Colistin utilization decreased by 60%, and the resistance of *Acinetobacter* spp. to colistin significantly decreased from 31% to 3% in a year. In addition, tigecycline utilization decreased by 46%. On the other hand, there were no significant changes in carbapenem utilization and resistance, which could be explained by switching from colistin and tigecycline to carbapenems.

**Conclusion:**

Adding an ID pharmacist and ID assistant consultants to the ASP team, and the strict restriction of colistin use was associated with significant reduction in colistin use and Acinetobacter resistance.

**Disclosures:**

**All Authors**: No reported disclosures

